# Alginate Lyases from Marine Bacteria: An Enzyme Ocean for Sustainable Future

**DOI:** 10.3390/molecules27113375

**Published:** 2022-05-24

**Authors:** Noora Barzkar, Ruilong Sheng, Muhammad Sohail, Saeid Tamadoni Jahromi, Olga Babich, Stanislav Sukhikh, Reza Nahavandi

**Affiliations:** 1 Department of Marine Biology, Faculty of Marine Science and Technology, University of Hormozgan, Bandar Abbas 3995, Iran; 2 CQM—Centro de Química da Madeira, Campus da Penteada, Universidade da Madeira, 9000-390 Funchal, Portugal; ruilong.sheng@staff.uma.pt; 3 Department of Radiology, Shanghai Tenth People’s Hospital, School of Medicine, Tongji University, Shanghai 200072, China; 4 Department of Microbiology, University of Karachi, Karachi 75270, Pakistan; msohail@uok.edu.pk; 5 Persian Gulf and Oman Sea Ecology Research Center, Iranian Fisheries Sciences Research Institute, Agricultural Research Education and Extension Organization (AREEO), Bandar Abbas 9145, Iran; stamadoni@gmail.com; 6 Institute of Living Systems, Immanuel Kant Baltic Federal University, A. Nevskogo Street 14, Kaliningrad 236016, Russia; olich.43@mail.ru (O.B.); stas-asp@mail.ru (S.S.); 7 Animal Science Research Institute of Iran (ASRI), Agricultural Research, Education and Extension Organization (AREEO), Karaj 8361, Iran; rnahavandi@gmail.com

**Keywords:** alginate, alginate oligosaccharides (AOs), alginate lyase, marine bacteria, brown algae, applications

## Abstract

The cell wall of brown algae contains alginate as a major constituent. This anionic polymer is a composite of β-d-mannuronate (M) and α-l-guluronate (G). Alginate can be degraded into oligosaccharides; both the polymer and its products exhibit antioxidative, antimicrobial, and immunomodulatory activities and, hence, find many commercial applications. Alginate is attacked by various enzymes, collectively termed alginate lyases, that degrade glycosidic bonds through β-elimination. Considering the abundance of brown algae in marine ecosystems, alginate is an important source of nutrients for marine organisms, and therefore, alginate lyases play a significant role in marine carbon recycling. Various marine microorganisms, particularly those that thrive in association with brown algae, have been reported as producers of alginate lyases. Conceivably, the marine-derived alginate lyases demonstrate salt tolerance, and many are activated in the presence of salts and, therefore, find applications in the food industry. Therefore, this review summarizes the structural and biochemical features of marine bacterial alginate lyases along with their applications. This comprehensive information can aid in the expansion of future prospects of alginate lyases.

## 1. Introduction

The marine ecosystem is considered the largest ecosystem, covering ~70% of this planet [1,2,3,4,5,6,7,8,9,10] and giving it a unique feature in the known universe. Reportedly, marine ecosystems make up the habitat of >80% of the living beings found on earth [11,12,13,14]. Among marine vegetation, brown algae (Phaeophyceae) holds a distinct position owing to its abundance [15]. Indeed, it plays a major role in CO_2_ removal and carbon storage for coastal regions [16].

Primarily, brown algae have a complex sugar composition, mainly including mannitol, laminarin, and alginate [17]. Mannitol is an alcohol derived from mannose, whereas laminarin is a polymer of β-1,3-linked glucose residues branched at 1,6-b [18,19]. Mannitol and laminarin are carbohydrate reserves that are accumulated by the algae during summer, and the content may reach 25–30% at the onset of autumn [20]. However, the major constituent of the brown algae polysaccharide repertoire is alginate [21], which makes up ~45% of the dry weight. There are various types of alginates according to the arrangement of monomers, β-d-mannuronate (M) and α-l-guluronate (G), either arranged in homopolymeric (polyM, polyG) or heteropolymeric (polyMG) fashion [22,23]. This anionic polymer serves as an important source of carbon for many marine microorganisms [21]. Commercially, alginates are extracted from different species of brown seaweeds, such as *Ascophyllum nodosum,* *Durvillaea potatorum*, *Ecklonia arborea*, *Ecklonia radiata*, *Laminaria digitata*, *Lessonia nigrescens*, *Laminaria hyperborea*, *Lessonia trabeculata*, *Macrocystis pyrifera*, *Saccharina japonica,* and *Sargassum* spp. [24,25].

Laminarin and mannitol are chemically less complex and, hence, can be converted by microbes into bioethanol, while alginates do not serve as a readily degradable carbon source. The structural complexity of alginates necessitates the activity of various lyases for its complete degradation; the enzymes are collectively called alginate lyases. These enzymes catalyze the degradation of glycosidic bonds through β-elimination [26] but vary in substrate specificity and, hence, are classified as polymannuronate (M) lyases, polyguluronate (G) lyases, and polyMG-specific lyases. The enzymes can also be distinguished on the basis of catalytic patterns as some act on the terminal residues (exo-enzymes) while others act randomly on the polymer chain (endo-enzymes). Studies on homology in amino acid sequences led to the classification of alginate lyases into polysaccharide lyase (PL) families. Structural elucidation revealed considerable heterogeneity, and the enzymes could be categorized into four groups, including β-jelly roll, (α/α)_n_ toroid, β-helix fold, or (α/α)_n_ toroid + β-jelly roll structures [27,28]. Alginate lyases are also diversified in terms of their molecular masses and grouped into large- (>60 kDa), medium (~40 kDa), and small sizes (25–30 kDa) [29].

Alginate lyases share tremendous applications with other industrial enzymes and are applied in agriculture, food, cosmetics, drug delivery, and biomedicine industries. Various organisms have been reported for the production of alginate lyases with varying substrate specificities. The enzyme producers include marine algae [30,31], marine mollusks [32,33], viruses (Chlorella virus) [34], fungi (*Corollospora intermedia*) [35], yeast (*Meyerozyma guilliermondii*) [36], and many terrestrial [29,37,38,39] and marine [40,41,42] bacteria. Nonetheless, bacteria are the far most important producers of alginate lyases. Considering the habitat and evolutionary history of the marine organisms, alginate lyases obtained from marine sources often exhibit remarkable salt tolerance and even salt activation [43,44,45,46]. *Vibrio harveyi*-28, a marine isolate, produced alginate lyase with a 24-fold increase in activity in the presence of 1 M NaCl [47]. Interestingly, some marine bacteria, such as *Pseudomonas aeruginosa* and *Azotobacter*, have the ability to produce alginate lyases, although they are incapable of utilizing alginate as a carbon source [48]. This review has collected the updated information about enzymatic and biochemical features and the applications of alginate lyases from marine bacteria.

## 2. Alginate and Alginate Lyases

Alginate is an abundant source of carbon in marine habitats. The cell wall of brown algae (Phaeophyceae) contains alginate, and since there are hundreds of species of brown algae, the material exists in large quantities. Some species, such as *Saccharin japonica* and *Undaria pinnatifda,* contain alginate that accounts for up to 45% of their dry weight [21]. Apart from brown algae, some species of bacteria produce alginate as a major component of extracellular polysaccharides or biofilms [49]. The bacterial alginate is constituted by the 1,4-glycoside bond-linked uronic acids, i.e., α-l-guluronic acid (G) and β-d-mannuronic acid (M) [50]. These basic units are arranged in different forms constituting three types of blocks, including poly α-l-guluronate (polyG), poly β-d-mannuronate (polyM), and their heteropolymer (polyGM), in which monomers are linked by α 1-4 glycosidic bond [51,52]. Alginate demonstrates various bioactivities and, hence, is widely employed in food and biomedicine industries. However, the applications are hindered by its high molecular weight, low water solubility, and unsatisfied bioavailability [53]. Degradation of alginate through chemicals (acid or alkali) or by physical process (such as microwave degradation) or through enzymatic action (by alginate lyases) yields alginate oligosaccharides with varying degrees of polymerization from 2 to 25. Owing to their high solubility and smaller molecular mass, alginate oligomers demonstrate different physiological activities, including antioxidative and immunomodulatory potential, have the capability of regulating blood sugar and blood lipids and can act as plant growth promoters [54,55].

The synthesis of alginate oligosaccharides by physical methods is energy extensive and can result in structural changes in the products. Enzymatic degradation methods are comparatively eco-friendly, energy-saving, and selective, and the products are biologically more active [56]. The enzyme-based methods employ the use of alginate lyases that catalyze β-elimination of glycosidic bonds. The enzymatic degradation of alginate yields various oligosaccharides, such as 4,5-unsaturated uronicresidues, mannuronate (ΔManUA), and guluronate (ΔGulUA) [57].

Alginate lyases vary in their substrate specificities depending on the amino acid sequence of the enzyme and the arrangement of monosaccharide residues in the substrate. Some lyases recognize mannuronate-containing substrates (PolyMlyases; EC 4.2.2.3), some can act on polymers of gulurunoate (PolyGlyases; EC 4.2.2.11), while some are capable of converting heteropolymers, i.e., (PolyMGlyases; EC 4.2.2.-) [56,58,59]. The action of these enzymes is utilized to determine the type of the polymer and to synthesize oligosaccharides of particular types. This is of particular interest as types of linkages between the substrate molecules (M-M, M-G, G-M, and G-G) can also be recognized by these enzymes [51]. These lyases can also be distinguished on the basis of catalytic patterns as exo-or endo-acting enzymes [60]. Exo-alginate lyases release monomers as the ultimate products, while endo-alginate lyases randomly degrade the polymer and mainly release a mixture of unsaturated oligosaccharides, including di-, tri-, and tetra-saccharides [61]. Based on the amino acid sequence alignment, alginate lyases can be classified into different polysaccharide lyase (PL) families, including PL5, PL6, PL7, PL8, PL14, PL15, PL17, PL18, PL31, PL32, PL34, PL36, PL39, and PL41 families, which are listed in the Carbohydrate-Active enzymes (CAZy) database (http://www.cazy.org/, accessed on 25 September 2021). Alginate lyases also exhibit a great variation in their structures, on the basis of which they are divided into four classes. The (α/α)_n_ toroid structure is mainly exhibited by the PL5 families, while the lyases in the PL6 and PL31 families share a β-helix fold. The β-jelly roll structure is found in alginate lyases of PL7, PL14, PL18, and PL36 families, and the (α/α)_n_ toroid + β-jelly roll architectures are adopted by the alginate lyases from the PL15, PL17, and PL39 [27,28]. Interestingly, architectures of several alginate lyases, particularly from the PL8, PL32, PL34, and PL41 families, are yet to be conclusively identified. Although all the types of alginate lyases catalyze the breaking of the glycosidic bond through β-elimination, they are categorized as metal ion-assisted β-elimination, Lys/Lys β-elimination, Tyr/Tyr β-elimination, His (Tyr′)/Tyr β elimination, and H_2_O-assisted β-elimination [28]. The great variation in the structure, substrate specificity, and mechanism of action of the alginate lyases is considered while designing its applications. It is worth noting that the enzymes provide efficient catalysts to produce oligosaccharides of variable length and different types under mild reaction conditions [62]. These functional oligosaccharides are in great demand [63,64,65], particularly when the raw material does not compete with the food resources [66,67]. The derivatization of the products of these enzymes has the potential to develop new and improved antibiotics with the emphasis on removing biofilms produced by pathogens such as *Pseudomonas* sp. [68].

## 3. Marine Sources of Alginate Lyase

In the past decades, alginate lyases have been isolated and purified from various marine organisms, including marine bacteria (*Pseudomonas* [69], *Vibrio* [70]), marine fungi [71], marine algae (*Laminaria*, *Saccharina* [72]), and marine mollusks (*Haliotis discushannai*) [59]. Inoue et al. identified a novel alginate lyase from the brown alga *Saccharina japonica* [31]. Alginate lyase activity has been detected within the extracts from several brown algae species, including *Laminaria digitata* [73], *Pelvetia canaliculata* [74], and *Undaria pinnatifida* [75], and has also been measured in the mid-gut gland of *Turbo cornutus* [76], the hepatopancreas of *Littorina* spp. [32] and *Dolabella auricola* [77], and the crystalline style of marine mussels *Choromytilus meridionalis* and *Perna perna* [78]. The alginate lyases secreted into the guts of various mollusks may facilitate the digestion process of devoured brown algal tissues. Furthermore, the largest variety of alginate lyases was discovered in marine bacteria, which served as the major sources [79]. For instance, Zhu et al. cloned an alginate lyase FsAlyPL6 from marine bacteria *Flammeovirga* sp. NJ-04 [80]. Zhu et al. reported that *Serratia marcescens* NJ-07 can produce alginate lyase [81]. Furthermore, the alginate lyase-producing marine bacteria are *Pseudomonas* sp. [82], *Photobacterium* sp. [83], *Vibrio* sp. [84], *Defluviitalea* *phaphyphila* [85], *Klebsiella aerogenes* type 25 [86], *Pseudomonas alginovora* XO17 [87], *Bacillus* sp. [42,88], *Corynebacterium* sp. ALY-1 [89], *Zobellia* *galactanivorans* [90], and *Agarivorans* sp. [91].

## 4. Alginate Lyase-Producing Marine Bacteria

Large quantities of alginates are produced by various algae in the ocean every year, they serve as nutrient resources for heterotrophic marine bacteria and, thus, play an ecological role in coastal ecosystems, similar to that of cellulosic and hemicellulosic biomass in terrestrial environments. Various alginate lyases produced by marine microbes play important roles in marine alginate degradation. A couple of alginate lyases were separated from different kinds of microorganisms in the past several years, especially from the bacteria on brown algae (such as *Bacillus* sp. obtained from rotten seaweed) [88], *Paenibacillus algicola* isolated from rotten brown algae samples collected from China [92], and *Pseudoalteromonas* sp. SM0524 separated from marine kelp residues [93]. Alginate-degrading bacteria were screened and identified from brown algae collected from a French beach and the Arctic region, which belonged to the classes Gamma-proteobacteria and Flavobacteria of the phylum Proteobacteria and Bacteroidetes [94,95]. Wang et al. (2017) reported that 12 different bacterial strains belonging to eight genera were recovered from the three brown algae (*Laminaria japonica*, *Sargassum horneri* and *Sargassum siliquastrum*) samples obtained from the coast of Nanhuangcheng Island, China, capable of excreting alginate lyases [25]. In addition, an alginate lyase-producing bacteria *Vibrio.* sp. QD-5 was isolated from rotten kelp [96]. Strain BP-2 producing the alginate lyase was screened and identified from rotted *Sargassum* collected from Weizhou Island, China [97].

## 5. Enzymatic Properties of Alginate Lyases from Marine Bacteria

Most of the marine-based alginate lyases are endolytic enzymes, which could break down glycosidic bonds of alginate and thus produce unsaturated oligosaccharides (Table 1). Endolytic alginate lyases were employed to prepare AOSs with various DPs. For example, Swift et al. discovered an endo-type alginate lyase AlgMsp from a marine bacterium *Microbulbifer* sp. 6532A, which produces AOSs DP2-5 [46]. Alg7D, an endo-type alginate lyase separated from *Saccharophagus degradans* 2-40^T^ mainly produced oligosaccharides with a DP of 3–5 [98]. It has been disclosed that depolymerized low DP alginate prepared through an enzymatic converter possesses various kinds of biological activities [63,99]. Nguyen et al. prepared a series of AOSs with the potential for efficient production of low DP alginate oligosaccharides by using a new marine actinobacterium-produced alginate lyase AlyDS44 *Streptomyces luridiscabiei* [100]. In addition, Aly-IV from *Vibrio.* sp. QD-5 [96] and AlgA from *Pseudomonas* sp. E03 [101] are two novel endolytic alginate lyase enzymes that can release a range of AOSs with low DP. In addition, a few exolytic alginate lyases could directly monomerize alginate to a monosaccharide [102] (Table 1). Interestingly, novel alginate lyases isolated from *Microbulbifer* sp. SH-1 [103] and BP-2 strain [97] demonstrated both exolytic and endolytic cleavage activities.

Substrate-specific alginate lyases are able to be utilized for determining sequences of alginate substrates and producing oligosaccharides with certain structures. The substrate specificities of these alginate lyases largely rely on their architectures, amino acid residues, and the alignment of the saccharide residues in the substrate. Various alginate lyases could recognize four different types of linkages, including G–G, M–M, G–M and M–G. The ALG-5 from *Streptomyces* sp. ALG-5 depolymerizes the polyG substrate [104]. The Alyw203 from *Vibrio* sp. W2 is also a polyG-specific alginate lyase [105]. High-alkaline alginate lyase, A1m, is a kind of mutant enzyme with cleavage specificity for the G–G linkage [91]. In addition, AlyPB2 from *Photobacterium* sp. FC615 specifically depolymerizes polyM [83]. However, there are several alginate lyases showing activities in both of them such as the lyases from *Vibrio* sp. QY108 [106], *Cobetia* sp. NAP1 [107], *Pseudoalteromonas* sp. SM0524 [93], *Pseudoalteromonas carrageenovora* ASY5 [108], *Agarivorans* sp. L11 [109], and *Streptomyces luridiscabiei* [100]. Moreover, bifunctional lyases possess different degradation activities toward different substrates. For instance, Aly-SJ02, a bifunctional alginate lyase from *Pseudoalteromonas* sp. SM0524, was preferable to depolymerizes poly (M) than poly (G) [93]. Aly-SJ02 showed lower K_m_ to polyG than that of polyM and sodium alginate [93]. Belik et al. reported a bifunctional endolytic alginate lyasesALFA3isolated from *Formosaalgae* KMM 3553^T^ [110]. These studies suggested that the bifunctional alginate lyases in alginate-utilizing bacteria could provide an efficient mechanism to utilize rich and reliable alginate sources for producing energy.

**Table 1:** Alginate lyases separated from various PL families of marine alginolytic bacteria.

Source	Localization	Substrate Specificity	Protein Name	Endo/Exolytic	PL	Main Products (DP)	Cleavage Site	References
*Photobacterium* sp. FC615	Extracellular	polyG	AlyPB1	endolytic	6		-	[83]
*Photobacterium* sp. FC615	Intracellular	polyM	AlyPB2	exolytic	15	-	-	[83]
*Vibrio* sp. QY108	-	polyM G	VsAly7D	exolytic	7	-	-	[106]
*Streptomyces* sp. ALG-5	Extracellular	polyG	ALG-5		7	-	-	[104]
*Cobetia* sp. NAP1	-	polyMG	AlgC-PL7		7	-	-	[107]
*Sphingomonas* sp.	-	polyMG	SALy	endolytic	7	3	G–G or G–M	[107]
*Flavobacterium* sp.	-	poly-(M)	FALy	endolytic	7	5–6	-	[111]
*Microbulbifer* sp. Q7.	Extracellular	polyG	AlyM	-	7	2–5	G–G or G–M	[112]
*Pseudoalteromonas* sp. SM0524	-	polyGM	Aly-SJ02	-	18	dimers and trimers from poly M, G3 and G4 from polyG	-	[93]
*Pseudoalteromonas* sp. SM0524	-	polyM	AlyPM	endolytic	7	dimers and trimers	-	[113]
*Microbulbifer* sp. 6532A	-	polyG	AlgMsp		7	2–5	-	[46]
BP-2 strain	-	polyM	Alg17B	endolytic and exolytic	17	2–6	-	[97]
*Vibriofurnissii* H1	-	polyGM	AlyH1		7	2–4	-	[114]
*Pseudoalteromonascarrageenovora* ASY5	extracellular	polyGM	Aly1281	endolytic	7	2	-	[108]
*Pseudoalteromonascarrageenovora* ASY5	extracellular	polyM	Alg823	endolytic	6	2	-	[115]
*Agarivorans* sp. L11	-	polyGM	AlyL1	endolytic	7	2–4	-	[109]
*Streptomycesluridiscabiei*	-	polyGM	AlyDS44	endolytic	7	2–4	-	[100]
*Formosaalgae* KMM 3553^T^	-	polyM	ALFA3	endolytic	7	1–20	M–M, M–G, G–M	[110]
*Formosaalgae* KMM 3553^T^	-	polyGM	ALFA4	endolytic	6	1–20	M–M	[110]
*Vibrio.* sp. QD-5	-	polyG	Aly-IV	endolytic	7	1–3	-	[96]
*Zobelliagalactanivorans*	Intracellular	poly-MG	AlyA1	endolytic	7	4–20	G–M, G–G	[90]
*Zobelliagalactanivorans*	Intracellular	polyG	AlyA5	exolytic	7	-	M–M, M–G, G–G	[90]
*Glaciecolachathamensis* S18K6T	-	polyG	AlyGC	-	6	-	-	[116]
*Vibrio* sp. W2	-	polyG	Alyw203	endo-type	7	1–2	-	[105]

According to the amino acid sequence and structural features, alginate lyases could be classified into several polysaccharide lyase (PL) families. As indicated in Table 1, marine bacteria-based alginate lyases are mainly PL6 and PL7 family members, which are endolytic. Moreover, alginate lyases are grouped into families based on the three-dimensional structures, which makes it possible to research the relationship between structure and function. The parallel β-helix family includes VsAly7D from *Vibrio* sp. QY108 [106], which belongs to the PL-7 family and AlyGC from *Glaciecola chathamensis* S18K6T [116], which belongs to the PL6 family, while the jelly-roll family includes Aly-SJ02 from *Pseudoalteromonas* sp. SM0524 of PL18 [117] and AlyA5 and AlyA1 from *Zobellia galactanivorans* of the PL-7 family [90].

Notably, some alginate-degrading strains could produce several alginate lyases to synergistically degrade exogenous alginate. The *Pseudoalteromonas* sp. strain ASY5 generates two extracellular alginate lyases Alg823 and Aly1281 (Table 1), which have similar action mode and main degradation products but different specificities to substrate. Although Alg823 andAly1281 are both bifunctional, Alg823 demonstrates the highest activity with polyM [68], while Aly1281 shows higher activity with polyG than that of polyM [108]. The similar action modes and main degradation products may bring them maximum enzyme activity under the same environmental conditions, and the substrate specificity difference leads to a synergistic alginate degradation effect of Alg823 and Aly1281. *Photobacterium* sp. FC615 produces extracellular (AlyPB1) and intracellular (AlyPB2) alginate lyases. Two alginate lyases have different substrate specificities, families, and modes of action. AlyPB1 is an alginate lyase with a preference for polyG, and AlyPB2 is a bifunctional lyase [83]. *Pseudoalteromonas* sp. 0524 secrets two extracellular alginate lyases (AlyPM and Aly-SJ02), which have different substrate specificities and, thus, synergistically facilitate the alginate degradation [93,113]. Additionally, *Formosa algae* KMM 3553^T^ secretes two endolytic alginate lyases (ALFA3 and ALFA4) with different substrate specificities. ALFA3 is a bifunctional lyase, while ALFA4 degrades only mannuronate blocks [110]. *Zobellia galactanivorans* produce two intracellular alginate lyases (AlyA1PL_7_ and AlyA5) with different modes of action [90].

## 6. Biochemical Properties of Marine Bacteria-Produced Alginate Lyases

There are some characteristics of alginate lyases produced from marine bacteria that are shown in Table 2. The optimal working conditions for most of the alginate lyases (especially the PL7 enzyme family) are between pH 7.0 and 8.5. Additionally, several alginate lyases exhibit the optimal activities in alkaline (Alyw203 from *Vibrio* sp. W2 [105]) and acidic (ALFA3 from *Formosa algae* KMM 3553^T^ [110] and SALy from *Sphingomonas* sp. [107]) environments (Table 2). Lyase Alyw202 has an optimal pH of 9.0, while the optimum pH value for lyases AlyM, AlyA1PL7, and AlyA5 is 7.0. The optimal pH of AlgMsp, AlyPB1, AlyPB2, ALG-5, AlgC-PL7, Aly1281, Alg823, and ALFA4 at pH 8.0 is between those values (Table 2). In addition, VsAly7D from *Vibrio* sp. QY108 showed its maximum activity at a pH of 8.0, and the enzyme stability remained within the pH range of 8.0 to 10.0. Therefore, VsAly7D works as an alkaline-stable alginate lyase that is generally stored under weak alkaline conditions and adapts different environments [106]. AlyPM showed the maximum activity at pH 8.5 and maintained ~70% of the maximum activity from pH 7.0 to 9.5 [113]. AlgC-PL7 retained ~50% of its maximum activity from pH 6 to 9. These results indicated that AlgC-PL7 generally possesses optimal activity under neutral conditions [107]. AlySJ-02 from *Pseudoalteromonas* sp. SM0524 demonstrated maximal activity at pH 8.5 and retained >50% activity at pH 7.0–10 after 20 min incubation [93]. Cold-adapted alginate lyase AlyL1 from *Agarivorans* sp. L11 showed the highest activity at a pH of 8.6 and maintained its stability from a pH of 6.0 to 9.6 [109].

As shown in Table 2, the optimal temperature for AlyPB1 from *Photobacterium* sp. FC615 [83], ALG-5 from *Streptomyces* sp. ALG-5 [104], AlyPM from *Pseudoalteromonas* sp. SM0524 [113], ALFA4 from *Formosa algae* KMM 3553^T^ [110], and AlyA1PL7 from *Zobellia galactanivorans* [90] is 30 °C. Alginate lyase produced by *Vibrio furnissii* H1 (AlyH1) [114] and *Agarivorans* sp. L11 (AlyL1) [109] works under a higher optimum temperature at 40 °C. Higher optimal temperatures were found on several alginate lyases produced by *Cobetia* sp. NAP1 (AlgC-PL7) [107], *Streptomyces luridiscabiei* (AlyDS44) [100], *Vibrio* sp. W2 (Alyw202 and Alyw203) [105,119], which had the optimum working temperature of 45 °C. The optimal temperature for Aly1281 from *Pseudoalteromonas carrageenovora* ASY5 [108], AlgMsp from *Microbulbifer* sp. 6532A [46], and Aly-SJ02 from *Pseudoalteromonas* sp. SM0524 [93] are around 50 °C. The highest optimum temperature of 55 °C was observed on alginate lyases produced by *Microbulbifer* sp. Q7. (AlyM) [112] and *Pseudoalteromonas carrageenovora* ASY5 (Alg823) [115]. Although most of the marine bacterial alginate lyases demonstrate an optimum temperature in the range of 30–55 °C, the alginate lyase isolated from *Photobacterium* sp. FC615 depicts optimal activity at 20 °C [83]. In addition, AlyL1 isolated from *Agarivorans* sp. L11 exhibited 54.5% and 72.1% of the maximal activity at 15 °C and 20 °C, respectively, suggesting that AlyL1 was a cold-adapted alginate lyase [109]. Alg17B exhibited different activity at 40–45 °C, and it has 90% of the maximum activity at 40 °C while only 10% of its activity remained at 45 °C; however, Alg17B has good thermal stability at 25–35 °C and maintained 80% of its enzyme activity within this temperature range. It could be seen that, with the temperature increase of 40 to 45 °C, the stability of Alg17B drastically diminished. Alyw203 alginate lyase possessed the maximum activity at 45 °C and the activity remained >80% in the range 40–55 °C [97]. AlyH1 showed high stability below 30 °C, and >60% of its activity could be maintained after incubation at 40 °C for 30 min [114].

**Figure 1:**
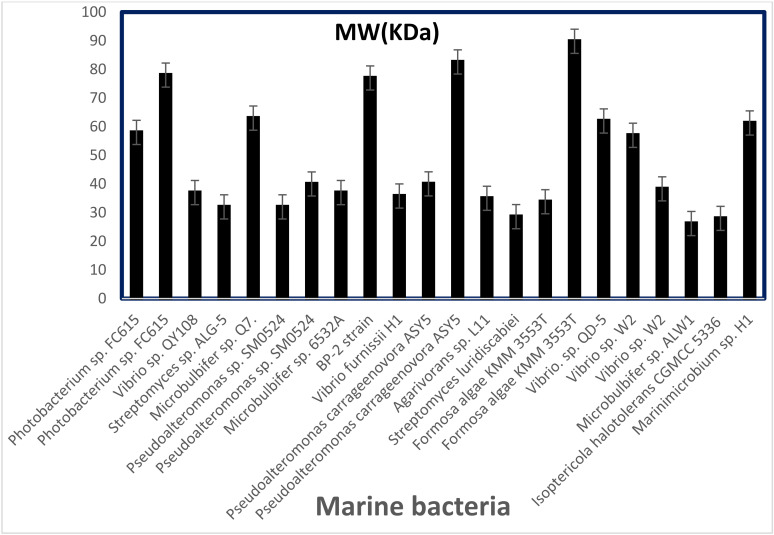
Molecular weight distributions of various alginate lyases produced by marine bacteria.

It could be noticed that various alginate lyases from different marine biological sources have different molecular weights. Generally, the molecular weight of alginate lyases produced by marine bacteria ranges from 24 to 110 kDa [120]. From an SDS–PAGE analysis, the molecular weight of alginate lyase *Vibrio* sp. QY108 was estimated to be 37 kDa [106]. AlyDS44 has a molecular weight of 28.6 kDa, which belongs to the low molecular weight (25–30 kDa) group of alginate lyases [51]. The alginate lyase produced by *Microbulbifer* sp. ALW1 has a molecular weight of 26.2 kDa [43]. Similar molecular weights were also observed on the alginate lyase extracted from *Isoptericola halotolerans* CGMCC 5336 (28 kDa) [44] and *Streptomyce* sp. ALG-5 (27.5 kDa) [104]. There are several high molecular weights alginate lyases, including AlyM from *Microbulbifer* sp. Q7 (63 kDa) [112], AlyA5 from *Zobellia galactanivorans* (69.5 kDa) [90] and AlgH1 from *Marinimicrobium* sp. H1 (61.3 kDa) [121]. The endolytic alginate lyases, such as ALFA4 from *Formosa algae* KMM3553^T^ and Alg823 from *Pseudoalteromonas carrageenovora* ASY5, possess a high molecular weight as well (Figure 1).

The effects of various cationic/anionic chemical species on alginate lyases enzyme activity are shown in Table 2. Usually, enzyme activity is influenced under the condition of divalent cations, which act as cofactors for increasing/inhibiting enzyme activity by inducing protein conformation change, replacing other enzyme cofactors, and alternating enzyme stability. Ca^2+^ and Mg^2+^ are stimulatory cofactors for regulating the enzyme activity of alginate lyases [60]. As shown in Table 2, in the presence of Mn^2+^ and Co^2+^, the alginate degradation activity of AlyDS44 increased by 242% and 219%, respectively, while Ca^2+^ and Mg^2+^ showed no effect on the AlyDS44 activities; however, Zn^2+^, Cu^2+^, and Fe^3+^ exhibited a slight or moderate enzyme inhibition effect [100]. For Aly-IV, its activity was significantly inhibited by Ba^2+^, Al^3+^, Ni^2+^, Zn^2+^, and Pb^2+^ (1 mM) but was promoted by Ca^2+^ (1 mM), K^+^ (5 mM) and Mg^2+^ (10 mM) [96]. The AlyH1 activity was inhibited by Fe^2+^, Cu^2+^, Zn^2+^, and Mn^2+^ but stimulated by Mg^2+^ (119.25%) and K^+^ (110.31%) [114]. Moreover, enzyme activities of AlyPB1 and AlyPB2 could be largely inhibited by Mn^2+^, Ni^2+^, Cu^2+^, Zn^2+^, Hg^2+^, and SDS. Additionally, AlyPB2 was also inhibited by Ag^+^ and Mg^2+^, but AlyPB1 was not inhibited by them. It could be noticed that, in the presence of Co^2+^, DTT, and β-mercaptoethanol, AlyPB2’s activity was increased to 158%, 186%, and 366%, respectively. Compared to AlyPB2, these chemicals did not significantly influence the activity of AlyPB1, which was strongly inhibited by Co^2+^ (10 mM) [83]. The metal cations such as Na^+^, K^+^, Mg^2+^, Ca^2+^, Ba^2+^, Ni^2+^, Co^2+^, and Sr^2+^ could improve Aly-SJ02’s enzyme activity and Zn^2+^ showed no effect, while Cu^2+^ and Sn^2+^ could slightly inhibit the activity of Aly-SJ02. In addition, EDTA (1 mM) could decrease the Aly-SJ02 activity to 48.3% [93]. The metal ion’s effect on the activity of AlyPM indicated that Ni^2+^ (2 and 10 mM) could inhibit its activity by ~50%. Cu^2+^ and Co^2+^ could increase the enzyme activity at a low concentration of 2 mM but inhibit the activity at a higher concentration of 10 mM. However, other metal ions (Mg^2+^, Ca^2+^, Ba^2+^, and Mn^2+^), had a negligible or low activation effect [113].

## 7. Enzyme Kinetics of Alginate Lyases from Marine Bacteria

Enzyme kinetics is an essential factor in evaluating the catalytic capability of an enzyme toward practical applications. However, since the alginate substrate is biochemically heterogeneous and alginates produced by various seaweeds have different mannuronic/guluronic (M/G) ratios, the enzyme kinetics of alginate lyases was difficult to measure. Additionally, the polyM, polyG, and polyMG subdomains and their frequencies are significantly different in different seaweed sources [60,122]. Alginate lyase-mediated production of alginate usually causes a mixture of polymers with different DP values, and their average length was determined by the preparation methodology and conditions. Therefore, it is hard to compare the enzyme kinetics among different alginate lyases [46]. The kinetic parameters of marine bacteria-based alginate lyases towards different substrates are shown in Table 3. For instance, with the substrate sodium alginate, the K_m_ and V_max_ of AlyH1 were measured as 2.28 mg/mL and 2.81 U/mg, respectively, indicating that AlyH1 (under sodium alginate substrate) possesses high enzyme efficiency [114]. Zhang et al. (2020) investigated the salt effect (NaCl: 300 mM; KCl: 1000 mM) on the enzyme kinetics of Aly1281 (substrate: sodium alginate), and it was found that adding 300 and 1000 mM of NaCl could decrease the K_m_ value by 54.9% and 74.3%, respectively. Compared to the K_m_ values under electrolyte-free conditions, the result indicated that the affinity of substrate and catalytic activity of alginate lyases could be greatly enhanced by adding salts or electrolytes, which is the salt-activation effect [108]. AlgMsp from *Microbulbifer* sp. 6532A showed a K_m_ of 3.4 mM for alginate [46]. Additionally, the catalytic efficiency (k_cat_/K_m_) of AlyL1 to alginate was calculated as 9952.8 ± 33.1 mg mL^−1^ s^−1^. AlyL1 exhibits a K_m_ value of 0.19 ± 0.04 mg/mL with a V_max_ value of 907.8 ± 72.5 U/mg protein. The results suggested that AlyL1 possesses a high affinity to alginate and could efficiently degrade alginates into oligosaccharides [109]. Moreover, K_m_ values of AlyA1 (PL7 family) from *Zobellia galactanivorans* with various seaweed alginate substrates range from 1.7 to 6.2 mM, with increased binding affinity to alginate with higher guluronate composition [90]. In addition, Aly-SJ02, an alginate lyase from *Pseudoalteromonas* sp. SM0524, has a higher K_m_ of 6.1 mM towards the alginate [93]. For seaweed-intake marine organisms, the low binding affinity of alginate lyases is acceptable due to the high concentration of alginate contents in seaweed (e.g., 17–45% *w/w* of dried brown seaweed) [21]. There are some notable exceptions of alginate lyases with K_m_ values in the micromolar range. Alginate lyases from different marine sources could have different polyM, polyG, or polyMG substrate specificities [60]. Typically, some alginate lyases prefer one substrate but still cleave the other substrates at a reduced rate. For example, Aly-SJ02, an alginate lyase from *Pseudoalteromonas* sp SM0524, degrades polyG and polyM with polyG-specific activity and 75% of that against polyM [93]. Additionally, the K_m_ and k_cat_/K_m_ of VsAly7D to alginate were calculated as 0.217 mM and 227 L mol^−1^ s^−1^, respectively [106]. Bifunctional alginate lyases could degrade different types of alginates, making them potential biocatalysts for industrial application.

## 8. Application of Alginate Lyases from Marine Bacteria

### 8.1. Preparation of AOs

Alginate oligosaccharides (AOs) possess various biological properties that provide benefits for improving human health. Their bioactivities, including antitumor [128], antidiabetic [129], antihypertensive [130], anti-inflammatory [131,132], antimicrobial [133], antioxidant [134], anticancer [99], immunomodulatory [135,136] and anti-radiation [43,137] properties, have been comprehensively summarized. Generally, traditional preparation methods for the production of AOs are usually under strong acidic and alkaline conditions [138], thus resulting in severe environmental damage. In contrast, enzyme-based AOs production methods are more “green” and environmentally sustainable. AOs prepared by enzymatic degradation methods showed special bioactivities due to their unsaturated oligosaccharide structures [139,140]. However, there is only one commercially available alginate lyase (CAS number: 9024-15-1, Sigma-Aldrich, St. Louis, MO, USA) with a high pH tolerance, high catalytic activity (>10,000 U/g) and magnificent heat stability, which is expensive and only sold in the form of reagents, and most of the marine bacterial-produced alginate lyases are just investigated at the laboratory level. NitAly obtained from *Nitratiruptor* sp. SB155-2 shows the highest alginate lyase activity at 70 °C [141], while alginate lyase Aly-IV (PL7 family) from *Vibrio.* sp. QD-5 [96] and Aly08 from *Vibrio* sp. SY01 [142] are alkaline-stable, with optimal working pH values of 8.9 and 8.35, respectively.

Apart from the above-mentioned pH and thermo-stable alginate lyases, several alginate lyases demonstrated great potential for producing alginate oligomers with various DPs. Since the bioactivities of AOs are largely dependent on their DP values and chemical structures [143,144], endolytic alginate lyase-produced oligosaccharides with various DPs and diverse structures have attracted significant attention. The investigations of new AOs-producing alginate lyases were mostly conducted at the laboratory scale, and it could be seen that the endolytic alginate lyase generally produced alginate oligomers with DPs ranging from 2 to 5 [144]. For instance, the alginate lyase isolated from *Isoptericola halotolerans* CGMCC 5336, purified by gel column chromatography and characterized by TLC and ESI-MS, could perform an elimination reaction on guluronic acid (active sites: G or G-Gresidues) and generate oligomers with DPs of 2–4 [145] (Table 4). Alg2A, an endolytic alginate lyase from *Flavobacterium* sp. S20, can produce oligosaccharides with high yields along with high DP values (e.g., DP5 (penta-), DP6 (hexa-) and DP7 (hepta-)saccharides) [146] (Table 4). Zhu et al. degraded alginate with alginate lyase from *Flammeovirga* sp. NJ–04 to prepare oligosaccharides with DP2-4 [58] (Table 4).

Notably, the combination of some endolytic and exolytic lyases could lead to a remarkable synergistic effect on the degradation of alginate. For AOs preparation, the simultaneous application of endolytic lyase AlyPB1 and exolytic lyase AlyPB2 could lead to significantly increased conversion from alginate to unsaturated monosaccharides, which could reach approximately seven-fold that of single AlyPB2 [83] (Table 4). Moreover, substrate-specific alginate lyases could be employed for the preparation of oligosaccharides with a specific molecular structure. Anne et al. constructed a diguluronic acid linkage-cleavable alginate lyase, which could be employed for the preparation of guluronic acid oligosaccharide [147]. Zhu et al. isolated a novel polyM-specific alginate lyase AlgNJ-07 from *Serratia marcescens* NJ-07, which showed good PolyM-degradation efficiency [81] and thus could act as a potential tool for the production of mannuronic acid oligosaccharide (Table 4).

**Table 4:** Some applications of alginate lyase from marine bacteria.

Enzyme	Source	Application	References	Field of Application
ALFA3	*Formosa algae* KMM 3553^T^	Preparation of alginate oligosaccharides	[110]	in agriculture, in feed production, to lower cholesterol levels in blood plasma
Aly1281	*Pseudoalteromonascarrageenovora* ASY5	Preparation of alginate oligosaccharides	[108]	in agriculture, feed production
AlgNJ-07	*Serratia marcescens* NJ-07	Preparation of alginate oligosaccharides	[81]	antimicrobials
AlgNJ-07	*Serratia marcescens* NJ-07	Preparation of alginate oligosaccharides	[81]	antimicrobials for the treatment of cystic fibrosis, in agriculture, in feed production, in medicine for the diagnosis of diseases, to lower cholesterol in blood plasma
FsAlgB	*Flammeovirga* sp. NJ–04	Preparation of alginate oligosaccharides	[58]	in medicine for the diagnosis
Aly	*Pseudomonas* sp. HZJ 216	Preparation of alginate oligosaccharides	[148]	antimicrobials, in medicine for the diagnosis of diseases
Alg2A	*Flavobacterium* sp. S20	Preparation of alginate oligosaccharides	[146]	to lower plasma cholesterol levels
Aly5	*Flammeovirga* sp. Strain MY04	Preparation of alginate oligosaccharides	[149]	in medicine for the diagnosis
AlyPB1 and AlyPB2	*Photobacterium* sp. FC615	Preparation of unsaturated monosaccharide	[83]	antimicrobials for the treatment of cystic fibrosis
Alg7A	*Vibrio* sp. W13	Preparation of alginate oligosaccharides	[144]	inhibition of lipid oxidation in food emulsions
Alginate lyase	*Isoptericolahalotolerans* CGMCC 5336	Preparation of alginate oligosaccharides	[145]	in feed production
AlyP1400	*Pseudoalteromonas* sp. 1400	The degradation of biofilms	[150]	in biofuel production
AlyL1	*Agarivorans* sp. L11	Produce TPC for bioenergy production	[151]	inhibition of lipid oxidation in industrial emulsions
Alg7D	*Saccharophagusdegradans* 2–40^T^	Produce DEH for bioenergy production	[123]	inhibition of lipid oxidation in industrial emulsions
AlyPB2	*Photobacterium* sp. FC615	Alginate Sequencing	[83]	in the production of alginates
Aly SM0524	*Pseudoalteromonas* sp. SM0524	Preparation of bioethanol	[152]	antimicrobials for the treatment of cystic fibrosis, for lowering plasma cholesterol levels
Alg17C	*Cobetia* sp. NAP1	Biofuels and chemicals production	[107]	in agriculture
Alginate lyase	*Shewanella* sp. Kz7	Biofuel production	[153]	in agriculture
Alginate lyase	*Gracilibacillus* sp. A7	Disposal of seaweed waste	[154]	in agriculture

### 8.2. Anti-Biofilm Activity

It is difficult for normal antibiotics to kill some pathogenic bacteria with complex biofilms on their surfaces. It was disclosed that alginate components in the biofilm of *Pseudomonas aeruginosa* could protect them from being recognized and cleared by the immune system and resisting antibiotic treatment [124,155]. Therefore, using a purified alginate lyase-antibiotic complex to synergistically treat *Pseudomonas aeruginosa* infections is a possible therapeutic method [125,156]. Recently, a purified alginate lyase (AlyP1400) from a marine *Pseudoalteromonas* sp. 1400 bacterium demonstrated the capability of disrupting the formation of biofilms of *Pseudomonas aeruginosa* by decomposing alginate within the extracellular polysaccharide matrix and thus enhancing the bactericidal activity of tobramycin, which may act as a promising strategy for combinational therapy [150] (Table 4).

### 8.3. Bioethanol Production

The alginate lyases are also employed as a potential tool for producing bioethanol. The exo-type alginate lyase depolymerizes the alginate oligomers into unsaturated monosaccharides and subsequently non-enzymatically converted to 4-deoxy-l-erythro-hexoseulose uronic acid (DEH), which was then reduced into 2-keto-3-deoxy-gluconate (KDG) by DEH reductase and was further connected to the Entner–Doudoroff (ED) pathway [157]. Normally, industrial microorganisms cannot directly utilize alginate as a starting resource to produce ethanol due to the lack of an alginate-mediated metabolic pathway. For a long time, it has been difficult to achieve efficient production of ethanol from brown algae. In 2012, Wargacki et al. [152] designed and prepared a bio-ethanol synthesis microbial platform using *E. coli* as a producer to secrete alginate lyase SM0524Aly from *Pseudoalteromonas* sp. SM0524 by an auto transporter (Table 4). Additionally, in *Vibrio splendidus* 12B01, an alginate lyase-encoding large gene cluster was introduced along with alginate catabolism-auxiliary gene clusters for achieving appropriate metabolism pathways. Finally, a pyruvate decarboxylase (Pdc) and an alcohol dehydrogenase B (AdhB)-encoding gene cluster was integrated into the *E. coli* chromosome to produce bioethanol. Moreover, endogenous *E. coli* genes, which encode fermentative byproducts, were removed. Accordingly, the fermentative yield of alginate, mannitol, and glucan could reach 0.28 g ethanol/per g dry brown algae (>80% of the maximum theoretical yield) [152]. Yagi et al. (2016) utilized Alg17C, an exo-oligoalginate lyase (PL7 family) isolated from halophilic Gram-negative bacterium *Cobetia* sp. NAP1 (brown algae *Padina arborescens* *Holmes*, as the bacterium resource) to depolymerize alginate into a monomeric sugar acid. Furthermore, Yagidis concluded that Alg17C could serve as the key enzyme to produce alginate monomers in the process of utilizing alginate for the production of biofuels and chemicals [107] (Table 4). It has been reported that the alginate lyase from *Shewanella* sp. Kz7 could degrade polyG blocks of alginate and accordingly produce monosaccharides such as 6-tetrahydroxy tetrahydro-2H-pyran-2-carboxylic acid (TPC), a useful intermediate for biofuel production [153] (Table 4).

### 8.4. Disposal of Seaweed Waste

In recent years, the amount of seaweed waste has drastically increased worldwide. One of the main organic components in seaweed is alginate, the content of which is as high as 50% in seaweed species such as wakame (*Undaria pinnatifida*) [158]. The disposal and re-utilization of seaweed waste are essential issues for the protection of marine environments and recycling of sustainable biomass. However, the degradation of alginate by general microorganisms is not easy to realize, mainly due to the complicated structures and molecular alignments of alginate. Thus, the isolation of specific microorganisms for alginate degradation is highly demanded, which is essential for the effective disposal of seaweed wastes. Tang et al. (2009) utilized alginate lyase-producing bacteria strain A7 (*Gracilibacillus* sp.) to degrade alginate in the wakame composting process. In a laboratory-scale test, after 72 h of composting, the alginate content in the wakame remarkably diminished from an initial value of 36.0% to 14.3%, suggesting the effectiveness of A7 for alginate decomposition [154] (Table 4).

### 8.5. Elucidate the Structure of Alginate

To profoundly understand the influence of the polymer architecture on the physico-chemical properties of alginate, alginate lyases have been utilized to analyze the fine polymer architecture, especially the alignment of α-l-guluronate (G) and β-d-mannuronate (M) units of alginate. It is also very necessary to investigate the fine architecture of alginate for the preparation of tailor-made alginate. Lu et al. combined ^1^H NMR spectroscopy with exolytic alginate lyase AlyPB2 to establish a method for sequencing alginate oligosaccharides [83] (Table 4). Compared with the traditional sequencing method, this method provides a simple strategy for characterizing the structure of alginate oligosaccharides.

**Figure 2:**
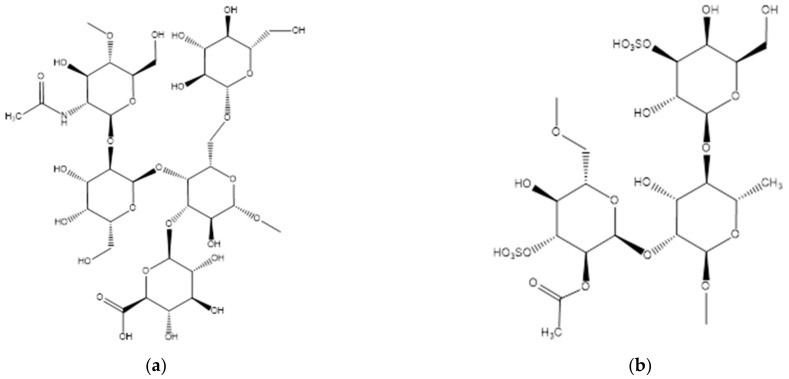

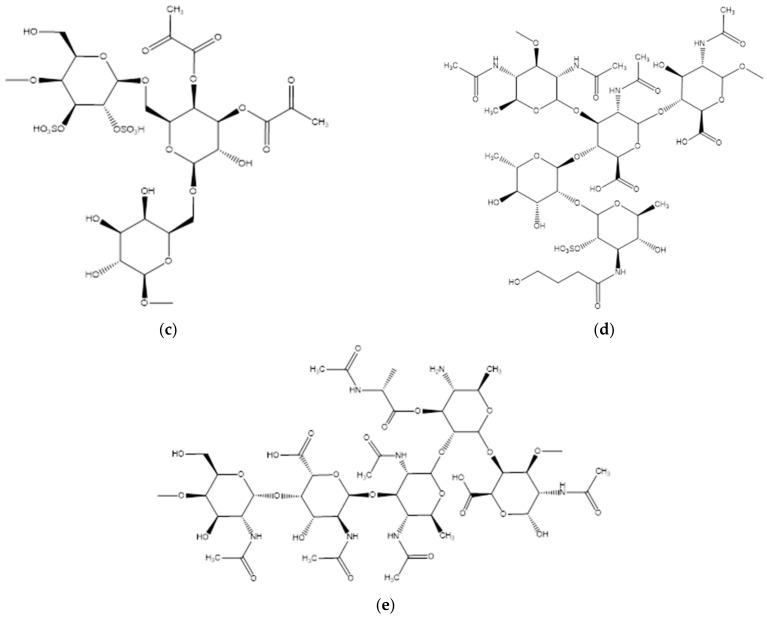
Alginate lyase structures of algae [159]. (**a**) Alginate lyase from *P. Algicola*; (**b**) alginate lyase from *L. Japonica*; (**c**) alginate lyase from *U. Pinnatifida*; (**d**) alginate lyase from *P. arborescens Holmes* (**e**) alginate lyase from *I. halotolerans*.

The O-antigenic polysaccharide of the *P. algicola* alga is composed of branched pentasaccharide repeating units containing monosaccharides quite common in nature (Figure 2a). *L. japonica* synthesizes a sulfated oligopolysaccharide composed of branched trisaccharide repeating units with the following structure: (Figure 2c). *S. horneri* also produces sulfated oligopolysaccharide, a galactan composed of linear trisaccharide repeat units and containing a pyruvic acid (Pyr) residue. *U. pinnatifida* produces a sulfated OPS composed of branched trisaccharide repeat units and has the following structure (Figure 2c). We isolated and analyzed another sulfated oligopolysaccharide from the *P. arborescens Holmes* alga. The repeating unit of the oligopolysaccharide of this algae is a branched pentasaccharide composed of the residues of 2,4-diacetamido-2,4,6-trideoxy-d-glucose (d-QuiNAc4NAc), l-rhamnose (l-Rha), 3-(4-hydroxybutyramido)-3,6-dideoxy-d-glucose, sulfated at the second position (b-d-Quip2SO3-3N(4Hb)), and two residues of 2-acetamido-2-deoxy-d-glucuronic acids (d-GlcpNAcA) (Figure 2d). *I. halotolerans* algae is an O-antigenic polysaccharide consisting of linear pentasaccharide repeating units containing residues of 2,4-diacetamido-2,4,6-trideoxy-Dglucose (d-QuiNAc4NAc), 2-acetamido-2-deoxy-d-galactose (d-GalNAc), 4-amino-4,6-dideoxy-d-glucose (d-Qui4N), N-acetyl-d-alanine (d-AlaAc), and two residues of 2-acetamido-2-deoxy-galacturonic acids (GalpNAcA) (Figure 2e) [159].

When studying the mechanism of action of alginate lyases, it was found that most of the studied alginate lyases function endolytically, i.e., they split the alginate molecules from the inside and do not produce significant amounts of oligomers at the beginning of the reaction [48]. If the reaction proceeds, the end products are typically dimers, trimers, tetramers, or pentamers [85]. However, several exoliases were described that remove single residues from the polymer end [48,160].

Gacesa [161] was the first to propose a reaction mechanism for alginate lyases. First, the negative charge on the carboxylate anion is shielded by the enzyme. This allows the proton to be abstracted from C-5. It is proposed to stabilize the intermediate enolate ion by resonance. Finally, electron transfer from the carboxyl group results in the formation of a double bond between C-4 and C-5 and cleavage of the O-glycosidic bond. It was found that cleavage is promoted by an amino acid residue acting as an acid [162]. The new non-reducing end will contain 4-deoxy-l-erythro-hex-4-enepyranosyluronate (Δ). This double bond is absorbed at 235 nm and is used to quantify alginate lyase activity [48]. The negative charge of most alginate lyases is stabilized by glutamine, arginine, or asparagine. It is important for the catalytic mechanism that, for M-residues, the C-5-proton and the departing oxygen on C-4 lie *syn* relative to each other, while for G residues they lie *anti* relative to each other. For the studied alginate lyases, it was found that for M-specific lyases, the C-5 proton is abstracted by tyrosine, which also acts as an acid facilitating the cleavage of the O-glycosidic bond. For lyases acting on G-residues, the C-5 proton is abstracted by histidine, while tyrosine again acts as an acid [162]. Alginate lyases belonging to the PL6 family do not follow this pattern. They use Ca^2+^ as a neutralizer, lysine as a proton abstracting residue, and arginine as an acid [162].

## 9. Conclusions Remarks

Thus, each year, various algae in the ocean produce large amounts of alginates, which serve as nutrient resources for heterotrophic marine bacteria and thus play an ecological role in coastal ecosystems similar to that of cellulose and hemicellulose biomass in terrestrial environments. Various alginate lyases produced by marine microbes have played an important role in the degradation of marine alginate, and several alginate lyases have been isolated from various types of microorganisms over the past few years, especially from brown algae bacteria. Alginate lyases derived from marine bacteria serve as a stable pool of enzymes in the process of alginate degradation and marine carbon utilization. Alginate lyases derived from marine bacteria have great potential for application in the pharmaceutical industry, biofuel production, and environmental protection. It is vital to discover more new alginate lyases and explore their structure, functions, and structure-function relationship in order to advance marine enzymology and biotechnology. Almost no alginate lyase product has been developed for therapeutic applications (such as antibacterial, anticancer, and other diseases). Based on the foregoing review, extensive research in the field of alginate lyases derived from marine bacteria in the direction of advanced biotechnologies is expected.

## Figures and Tables

**Table 2:** Biochemical properties of alginate lyase purified from marine alginolytic bacteria.

Source	Enzyme	Opt. pH	pH Stability	Opt. Temp (°C)	Thermal Stability	PI	Activators	Inhibitors	Gen Bank Accession No.	References
*Photobacterium* sp. FC615	AlyPB1	8.0	-	30	-	4.88	-	Hg^2+^, Ni^2+^, Mn^2+^, Zn^2+^, Cu^2+^, SDS, Co^2+^	MN116685	[83]
*Photobacterium* sp. FC615	AlyPB2	8.0	-	20	-	5.01	Co^2+^, DTT, β-mercaptoethanol	Hg^2+^, Ni^2+^, Mn^2+^, Zn^2+^, Cu^2+^, SDS, Ag^+^, Mg^2+^	MN116686	[83]
*Vibrio* sp. QY108	VsAly7D	7.6	stable at pH 7.6~10.6, stable at pH 9.0~10.0 (12 h, with 80% activity)	35	46.5% (20 °C) and 83.1% (30 °C) of the initial enzyme activities	5.65	-	Zn^2+^, Fe^3+^, Cu^2+^, SDS and EDTA	QPB15428	[106]
*Streptomyces* sp. ALG-5	ALG-5	8.0	-	30	-	-	-	-	EU137870	[104]
*Cobetia* sp. NAP1	AlgC-PL7	8.0	~50% lyase activity at pH 6~9.	45	>90% of the initial enzyme activity (heating at 70~80 °C for 15 min), 80% (heating at 90 °C for 15 min).	-	-	-	-	[107]
*Sphingomonas* sp.	SALy	6.5	-	-	70% of the initial enzyme activity at 55 °C	-	-	-	2CWS	[107]
*Flavobacterium* sp.	FALy	7.5	-	-	30–40% of the initial enzyme activity at 55 °C for 4 h; lost its activity at 60 °C	-	-	-	JF412659	[111]
*Microbulbifer* sp. Q7.	AlyM	7	-	55	32% of initial enzyme activity at 45 °C for 2 h; 14.7% at 55 °C for 1 h	4.4	K^+^, Ca^2+^, Mg^2+^, glycine	Zn^2+^, Cu^2+^, Li^+^, Fe^3+^, Fe^2+^, Mn^2+^, EDTA, SDS	WP066959628.1	[112]
*Pseudoalteromonas* sp. SM0524	Aly-SJ02	8.5	stable at pH 8.0 ~50% activity at pH 7.0–10 for 20 min	50	Remain stable for 41 min at 40 °C and 20 min at 50 °C		Na^+^, K^+^, Mg^2+^, Ca^2+^, Co^2+^, Ba^2+^, Ni^2+^, Sr^2+^	Cu^2+^, Sn^2+^, EDTA	EU548075	[93]
*Pseudoalteromonas* sp. SM0524	AlyPM	8.5	>70% of its highest activity at pH 7.0~9.5	30	19% of the highest activity at 5 °C. unstable at >30 °C low T_m_ at 37 °C.		Cu^2+^,Co^2+^	Ni^2+^	EU548076	[113]
*Microbulbifer* sp. 6532A	AlgMsp	8.0	-	50	activity down by 86 at 60 °C, no activity at 70 °C	-	-	Ni^2+^, Ca^2+^	AB603802	[46]
BP-2 strain	Alg17B	7.5–8.0	stable at pH 7.0–8.0, enzyme activity was reduced to 33% at pH 8.5	40–45	stable at 25–35 °C. 90% of the enzyme activity at 40 °C	-	Na^+^	Ca^2+^, Zn^2+^	MH820150.1	[97]
*Bacillus* sp.	-	8.0	stable at pH 4.0–9.0	50	stable at 45 °C. 50% at 50 °C for 105 min and maintain 100% activity at 45 °C after 180 min	-	Mg^2+^, Ca^2+^, K^+^	Zn^2+^, Co^2+^, Li^+^, EDTA, PMSF	LC457966	[88]
*Vibrio furnissii* H1	AlyH1	7.5	stable at pH 7.0–8.0 for 12 h, >60% activity at pH 6.5–8.5, 80% activity at pH 7.0–8.0	40	stable at <30 °C. >60% of activity at 40 °C for 30 min	-	Na^+^, Mg^2+^, K^+^	Zn^2+^, Fe^2+^, Cu^2+^, Mn^2+^, Ag^+^	MG214325	[114]
*Pseudoalteromonascarrageenovora* ASY5	Aly1281	8.0	>65% enzyme activity at pH 6.0–9.5. >70% of the enzyme activities at pH 7.0–9.0	50	>50% of the activity at 45–55 °C	9.06	-	-	-	[108]
*Pseudoalteromonascarrageenovora* ASY5	Alg823	8.0	>80% activity at pH 6.0–10.0 (4 °C for 24 h)	55	~75% of the optimal activity at 50 °C for 30 min	-	Mg^2+^, Ca^2+^, Na^+^, and K^+^	CTAB	-	[115]
*Agarivorans* sp. L11	AlyL1	8.6	stable at pH 6.0–9.6	40	54.5% and 72.1% of optimal activity at 15 °C and 20 °C, respectively	-	-	-	KM018274	[109]
*Streptomycesluridiscabiei*	AlyDS44	8.5	>70% of the maximum activity at pH 6.5–9.5.	45	>80% enzyme activity at 35 °C to 55 °C.	-	Mn^2+^, Co^2+^, Fe^2+^	Zn^2+^, Cu^2+^	OK169607	[100]
*Alteromonas* sp. H-4	-	7.5	stable at pH 6.6–9.0, <20% activity at pH < 5.0	30	20% and 40% decrease in the enzyme activity at 30 and 40° C for 5 min, respectively.	-	MnCl_2_ or BaCl_2_,	EDTA. Na^+^, ZnSO_4_, or CdCl_2_	-	[118]
*Formosaalgae* KMM 3553^T^	ALFA3	6.0	-	35	50% activity at 42 °C for 30 min	-	-	-	PRJNA299442	[110]
*Formosaalgae* KMM 3553^T^	ALFA4	8.0	-	30	stable up to 30 °C; 50% activity at 37 °C for 1 h 40 min.	-		-	PRJNA299442	[110]
*Vibrio.* sp. QD-5	Aly-IV	8.9	>80% activity at pH 7.0–10.0.	35	stable at <30 °C for 30 min	5.12	K^+^, Mg^2+^	Ba^2+^, Al^3+^, Ni^2+^, Zn^2+^, Pb^2+^, EDTA	PRJNA382465	[96]
*Zobelliagalactanivorans*	AlyA1	7.0	-	30	-	-	-	-	-	[90]
*Zobelliagalactanivorans*	AlyA5	7.0	-	-	-	-	-	-	-	[90]
*Vibrio* sp. W2	Alyw203	10	>80% of the highest activity at pH 4.0–10.0.	45	>90% of its initial activity at 10 °C for 20 min >80% activity at 40–55 °C	6.09	Fe^3+^, Cu^2+^, Zn^2+^, Al^3+^	SDS, EDTA		[105]
*Vibrio* sp. W2	Alyw202	9	>80% activity at pH 5.0–9.0 (4 °C) for 12 h, >60% activity at pH 3.0–10.0 (4 °C) for 12 h	45	-	5.10	Mn^2+^ and Co^2+^	Na^+^, Mg^2+^ and Ba^2+^, EDTA and SDS	-	[119]

**Table 3:** Kinetic parameters of alginate lyases from marine bacteria toward sodium alginate, polyM, and polyG.

Enzyme	Source	Substrate Preference	K_m_	V_max_	k_cat_	References
AlyPM	*Pseudoalteromonas* sp. SM0524	polyM	3.15 mg/mL (0.5 M NaCl) and 74.39 mg/mL (0 M NaCl) for sodium alginate	-	-	[113]
ALFA3	*Formosa algae* KMM 3553^T^	polyGM	0.12 ± 0.01 mg/mL	0.128 × 10^−3^ M/min for G, 0.150 × 10^−3^ M/min for MG, 0.211 × 10^−3^ M/min for M	3.52 s^−1^ for G, 4.13 s^−1^ for MG and 5.80 s^−1^ for M	[111]
ALFA4	*Formosa algae* KMM 3553^T^	polyM	3.01 ± 0.05 mg/mL for polyM	0.314 × 10^−3^ M/min for MG	2.88 s^−1^ for MG	[111]
ALW1	*Microbulbifer* sp. ALW1	-	1.03 mg/mL for sodium alginate	4.63 U/mg for sodium alginate	69.38 s^−1^ for sodium alginate	[42]
Aly1281	*Pseudoalteromonascarrageenovora* ASY5	-	0.3180 (0.3 M NaCl) and 0.1810 mg/mL (1.0 M NaCl), respectively, 0.2805 (0.3 M KCl) and 0.1631 (1.0 M KCl) for sodium alginate	-	2.185 s^−1^ (in 0.3 NaCl), 2.095 s^−1^ (in 1.0 M NaCl), 1.875 s^−1^ (in 0.3 KCl), 1.502 s^−1^ (in 1.0 M KCl) for sodium alginate	[123]
AlgNJ–07	*Serratia marcescens* NJ-07	-	0.53 mM for sodium alginate, 0.27 mM for polyM	74, 67 nmol/s for sodium alginate and polyM	34 for sodium alginate, and 31 s^−1^ for polyM	[81]
Aly-IV	*Vibrio.* sp. QD-5	-	0.2223 g/mL for sodium alginate, 0.3274 g/mL for polyG	3.6 OD_235_/h for sodium alginate, 2.8321 OD_235_/h for polyG	-	[97]
Aly-SJ02	*Pseudoalteromonas* sp. SM0524	bifunctional	1.086 for sodium alginate, 0.465 for polyG, 2.751 mg/mL for polyM,	8.074 OD_235_/h for sodium alginate, 5.318 OD_235_/h for polyG, 7.131 for polyM	-	[93]
Alg823	*Pseudoalteromonascarrageenovora* ASY5	-	0.15 mg/mL for sodium alginate	1.84 U/g for sodium alginate	1.19 × 10^6^ s^−1^ for sodium alginate	[93]
VsAly7D	*Vibrio* sp. QY108	-	0.217 mM for alginate	-	42.26 s^−1^ for sodium alginate	[107]
AlgM4	*Vibrio weizhoudaoensis* M0101	bifunctional	2.72 mg/mL, for sodium alginate	2.75 nmol/s for sodium alginate	30.25 s^−1^ for sodium alginate	[124]
AlgH	*Marinimicrobium* sp. H1	-	6.6 ± 2.2 mg·mL^−1^ for sodium alginate, 7.6 ± 1.6 mg·mL^−1^ for polyG, 9.1 ± 2.4 mg·mL^−1^ for polyM	224.6 ± 33.6, 146.6 ± 15.6, 62.6 ± 8.8 U/mg of protein, respectively, for sodium alginate, polyG and polyM	260.6 ± 36.2 s^−1^ for sodium alginate, 155.7 ± 17.1 s^−1^ for polyG, 66.8 ± 6.7 s^−1^ for polyM	[121]
AlyH1	*Vibrio furnissii* H1		2.28 mg/mL for sodium alginate	2.81 U/mg for sodium alginate	-	[115]
AlgNJU-03	*Vibrio* sp. NJU-03	bifunctional	8.50 mM for sodium alginate,, 10.94 mM for polyM, 4.00 mM for polyG	1.67 nmol/s for sodium alginate, 0.30 nmol/s for polyM, 2.50 nmol/s for polyG	30.64, 5.50, 45.87 s^−1^, respectively for sodium alginate, polyM and polyG	[125]
AlgNJ–04	*Vibrio* sp. NJ04	-	0.49 mM for alginate, 0.86 mM for polyM, 0.24 mM for polyG	72 pmol/s for alginate, 95 for polyM, 35 pmol/s for polyG	59 s^−1^ for alginate, 77 s^−1^ for polyM, 29 s^−1^ for polyG	[124]
Alys1	*Tamlana* sp. S12	polyM	0.20 ± 0.01 mM for sodium alginate	-	4.43 ± 0.027 s^−1^ for sodium alginate	[126]
AlyC3	*Psychromonas* sp. C-3	polyM	0.24 ± 0.05 mg/mL for polyM	19,704.73 ± 1865.49 U/mg of protein for polyM	-	[127]
AlgMsp	*Microbulbifer* sp. 6532A	polyG	3.46 ± 0.9 mM for alginate, 1.8 ± 0.4 mM for polyG, 6.8 ± 2.1 mM for polyM	5765, 3562, 6368 U/mg of protein for alginate, polyG and polyM, respectively	42 s^−1^ for alginate,26 s^−1^ for polyG, 46 s^−1^ for polyM	[46]
A1m	*Agarivorans* sp. JAM-A1m	-	-	38.4, 285.7, 416.7, and 526.3 U/mg of protein (0, 0.1, 0.2, and 0.5 M NaCl, respectively) for sodium alginate	-	[91]

## Data Availability

Not applicable.
